# Phlebotomine sand flies on the crossroads of Anatolia: transmitted diseases and vectors

**DOI:** 10.1186/1756-3305-7-S1-O21

**Published:** 2014-04-01

**Authors:** O Kasap Erisoz, A Belen, C Alkan, F Gunay, V Dvorak, K Ergunay, S Aydın, J Votypka, A-L Banuls, R Charrel, A Özkul, Y Özbel, P Volf, B Alten

**Affiliations:** 1 Faculty of Science, Hacettepe University, Department of Biology, Ecology Section, Beytepe-Ankara-Turkey; 2 Unite des Virus Emergents, Marseille Universite, UMR190 "Emergence des Pathologies Virales, Faculte de Medecine, Marseille, France; 3 Parasitology Department, Charles University, Faculty of Science, Prague, Czech Republic; 4 Department of Medical Microbiology, Hacettepe University, Faculty of Medicine, Virology Unit, Sihhiye-Ankara-Turkey; 5 Department of Communication Sciences, Hacettepe University, Faculty of Communication, Beytepe-Ankara-Turkey; 6 IRD (IRD 224-CNRS 5290-UM1-UM2), MIVEGEC, Montpellier, France; 7 Department of Virology, Ankara University, Faculty of Veterinary Medicine, Diskapi-Ankara-Turkey; 8 Parasitology Department, Ege University, Faculty of Medicine, Bornova, Izmir, Turkey

## 

The Western Palearctic (WP) is composed of Europe, Middle East and North Africa. In this territory, the Mediterranean Sea, and the land under the influence of the Mediterranean Sea is the most important geographical character for both migration and dispersion of organisms; especially for invertebrates including sand flies. Anatolia (Asia-Minor) takes place on the crossroads of this area and these events.

The phlebotomine sand flies (Diptera: Psychodidae, Phlebotominae) are vectors of several infectious pathogens causing leishmaniases and arbovirus infections due to phleboviruses. Several of these diseases have wide geographical distributions in the WP, and give rise to occasional epidemic outbreaks. In numerous countries, increasing risk factors are making sand fly-borne diseases a major public and veterinary health problem. Many studies on phylogenetic relationship among sand fly taxa, their distribution, population structure and diseases of phlebotomine species have been already published, but there are still many gaps waiting to be filled up in, especially, Anatolia. In this point, scientists have to discuss some deficiencies under cover of geography, history and phylogenetic studies to understand the mechanisms of distribution of both sand fly species and their pathogens in Anatolia.

In this presentation, updates in distribution of sand fly species with state of art maps of EU-VBORNET project, possible new species, leishmaniasis and phleboviruses epidemiology will be discussed with an emphasis on several studies performed by our group between 2000 and present in Anatolia.

Studies were supported by EU-FP7 Edenext project, HU-Scientific Research Foundation and Turkish Scientific and Technical Research Council.

